# Nano‐therapeutic strategies to target coronavirus

**DOI:** 10.1002/VIW.20200155

**Published:** 2021-03-09

**Authors:** Mohd Ahmar Rauf, Munazzah Tasleem, Ketki Bhise, Katyayani Tatiparti, Samaresh Sau, Arun K. Iyer

**Affiliations:** ^1^ Use‐inspired Biomaterials & Integrated Nano Delivery (U‐BiND) Systems Laboratory Department of Pharmaceutical Sciences Eugene Applebaum College of Pharmacy and Health Sciences Wayne State University Detroit Michigan; ^2^ Bioinformatics Infrastructure Facility, Department of Computer Science Jamia Millia Islamia University New Delhi 110025 India; ^3^ Molecular Imaging Program Barbara Ann Karmanos Cancer Institute Wayne State University School of Medicine Detroit Michigan

**Keywords:** 2019‐nCoV, coronavirus, COVID‐19, nanotechnology, SARS‐CoV‐2, vaccine development, viral diagnosis, viral pathogenesis, virus therapy

## Abstract

The coronaviruses have caused severe acute respiratory syndrome (SARS), the Middle East respiratory syndrome (MERS), and the more recent coronavirus pneumonia (COVID‐19). The global COVID‐19 pandemic requires urgent action to develop anti‐virals, new therapeutics, and vaccines. In this review, we discuss potential therapeutics including human recombinant ACE2 soluble, inflammatory cytokine inhibitors, and direct anti‐viral agents such as remdesivir and favipiravir, to limit their fatality. We also discuss the structure of the SARS‐CoV‐2, which is crucial to the timely development of therapeutics, and previous attempts to generate vaccines against SARS‐CoV and MERS‐CoV. Finally, we provide an overview of the role of nanotechnology in the development of therapeutics as well as in the diagnosis of the infection. This information is key for computational modeling and nanomedicine‐based new therapeutics by counteracting the variable proteins in the virus. Further, we also try to effectively share the latest information about many different aspects of COVID‐19 vaccine developments and possible management to further scientific endeavors.

## Graphical abstract

The role of nanotechnology in the detection and the therapeutics of the COVID‐19. The image was created with biorender.com.

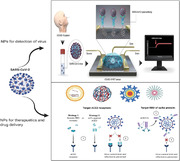

## 1 INTRODUCTION

The outbreak of the novel coronavirus disease COVID‐19 caused by the recent 2019‐CoV from the severe acute respiratory syndrome (SARS)‐COV2 virus family has spread rapidly. It represents a pandemic threat to the health of individuals around the globe. Even though the disease originated in Hubei province (Wuhan) of China, it has now spread to different countries with over 60 million confirmed cases and more than 1.42 million deaths which resulted in the global emergency declared by the World Health Organization (WHO).^1^ COVID‐19 mode of infection is similar to that of SARS‐CoV and Middle East respiratory syndrome (MERS)‐CoV, and it infects the human respiratory system and causes pneumonia.^2^ It is found to be more transmissible and contagious than SARS disease. However, a few reports also suggested that it may influence the gastrointestinal system and central nervous system and lead to multiple organ failure.^3^, ^4^, ^5^ Severe illnesses and deaths are seen among the elderly who are diagnosed with other chronic underlying conditions with about 14.8% mortality rate in those over 80 years old and 0.2% rate in those under 40 years old. In terms of addressing the pandemic, there are no vaccines currently available, although there is considerable work being done to develop one.^6^, ^7^ On March 16th, a volunteer in the United States was the first to receive a potential SARS‐CoV‐2 as part of a phase I safety trial. The time from sequencing the viral RNA to developing this prototype was only an unprecedented 42 days. In addition, there is presumably no preexisting immunity in the population against this novel coronavirus, and everyone in the community is assumed to be susceptible.

### 1.1 Structure and replication of the virus

Coronaviruses are relatively large viruses that belong to the coronavirinae subfamily in the Nidoviral group of Coronaviridae, which includes four genera: Alpha coronavirus, Beta coronavirus, Gamma coronavirus, and Delta coronavirus.^8^, ^9^, ^10^ CoVs have a positive‐sense RNA (+ ssRNA) genome (∼30 kb) with a 5′‐ cap and a 3′‐ poly‐A tail. The genomic RNA is used as a layout for directly translating polyprotein(pp)1a/1ab which encodes non‐structural proteins (nsps) to form a dual layer of the replication‐translation complex (RTC). A settled RNAs (sgRNAs) arrangement is subsequently synthesized by RTC.^11^ These subgenomic mRNAs have a regular arrangement of 5′‐open and 3′′. The termination of transcription leads to a positioning of a leading RNA at the translation regulatory sequences sequences between open read frames (ORFs). These short subgenomic RNAs are built to shape subgenomic mRNAs.^12^, ^13^, ^14^

**FIGURE 1:**
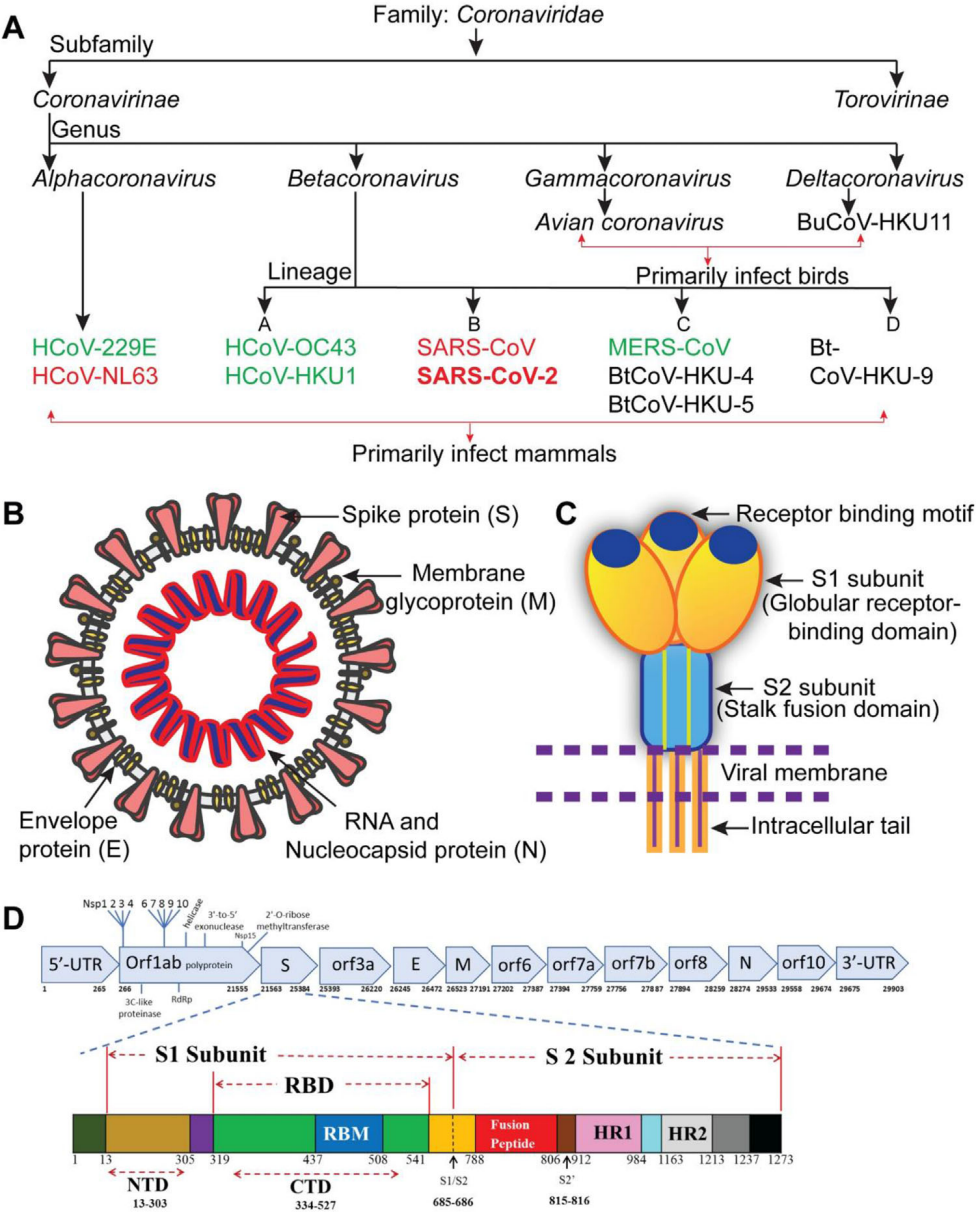
Coronavirus classification and structure. (A) Coronavirus classification: the seven recognized HCoVs are shown in green and red. Red bind HCoVs to the ACE2 host receptor. (B) The SARS‐CoV‐2 structure scheme; the “Desiree Ho,” an Innovative Genomics Institute "illustrator is adapted from the https://innovativegenomics.org/free-covid-19-illustrations/. (C) Cartoon illustrates main features and the SARS‐CoV‐2 S protein trimeric structure. (D) SARS‐CoV‐2 schemas (top) and S‐protein (bow); NCBI (NC 045512.2) and Expasy (https://covid-19.uniprot.org/uniprotkb/P0DTC2) schematic annotations have been updated respectively) (adapted from Mittal et al^15^ without modifications) Abbreviations: CTD, C‐terminal domain; E, envelope; HCoV, human coronavirus; HR1/2, heptad repeat 1/2; M, membrane; N, nuclear‐capside; NSP, nonstructural protein; NTD, n‐terminal domain; orf, open reading framework; RBD, receptor‐binding motif; RdRp, RNA‐dependent polymerase, protein, spike protein; SARS‐CoV‐2, severe acute respiratory syndrome

CoV genomes and subgenomes contain approximately six ORFs. The first ORF (ORF1a / b), about two‐thirds long, codes 16 non‐basic proteins (nsp1‐16) in addition to the nsp1‐necessary gamma coronavirus. Somewhere in the range of ORF1a and ORF1b, there is a 1‐frameshift which leads to producing two polypeptides: pp1a and pp1ab. These polypeptides are formed in 16 nsps by virally encoded chymotrypsin‐like proteases (3CLpro) or fundamental proteases (Mpro) and a few papain‐like proteases (PLPs). Different ORFs of the 33% near the 3′′ end encodes have four essential auxiliary proteins at all times: spike (S), sheet (M), envelope (E) and nucleocapsid (N) proteins^15^ [Figure 1]. Other than these, CoVs encode exceptional essential and fresh proteins, e.g. HE protein, 3a / b protein, 4a / b protein.^16^ The subgenomic RNAs of CoVs are deciphered from all essential structural proteins.^12^, ^17^

**FIGURE 2:**
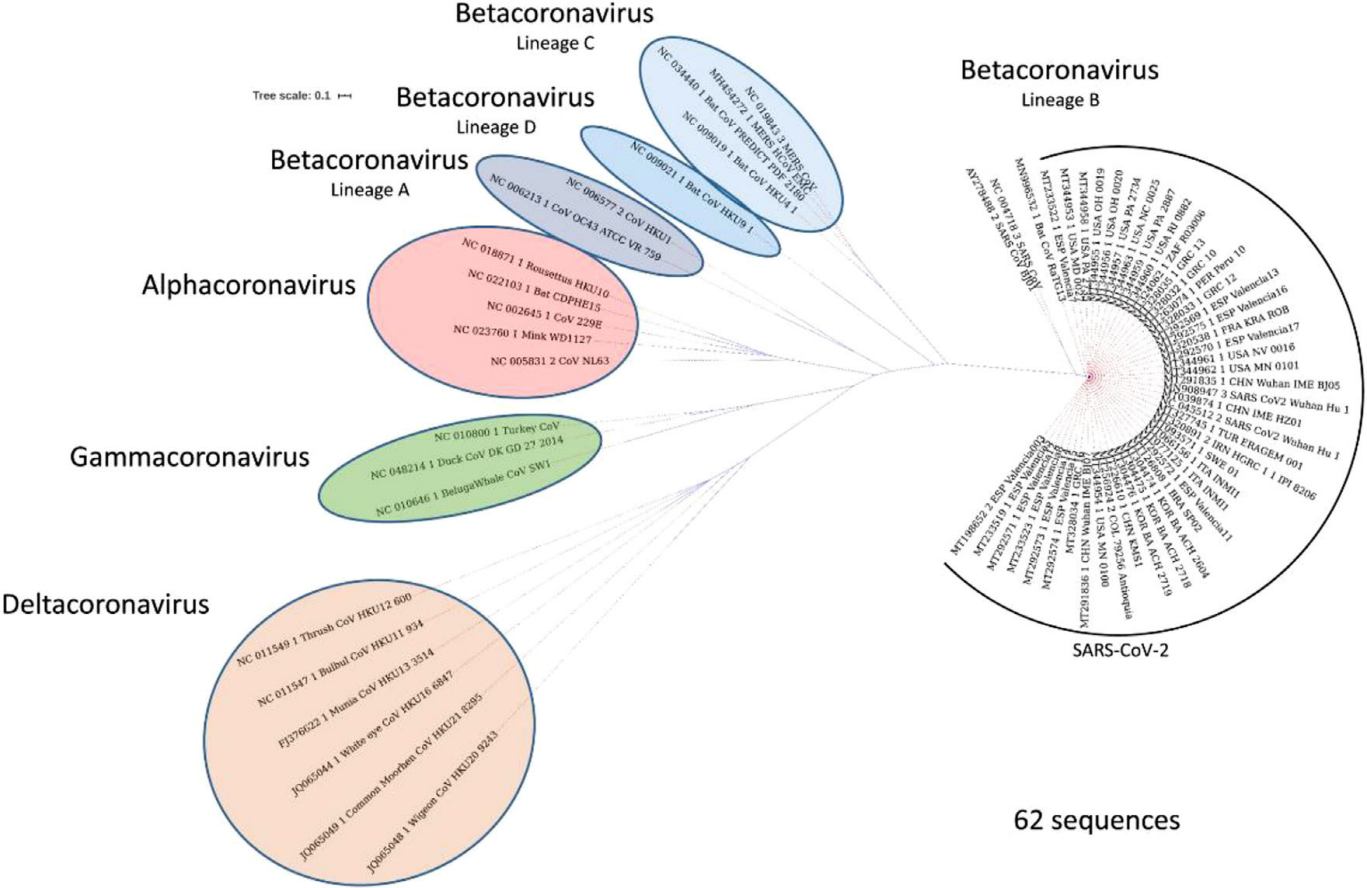
Phylogenetic relationships in the subfamily of coronavirinae. The Subfamily consists of four genera: alphacoronavirus, betacoronavirus, gamma coronavirus and delta coronavirus (lineages A, B, C, and D). Randomly, we have chosen 62 genome sequences for SARS‐CoV‐2, representing 15 countries with other members of the Coronavirinae subfamily. The NgPhylogeny.fr method was used to establish the phylogenetic tree. The study shows that SARS‐CoV‐2 is closely linked to it. It is also known as new member of the lineage B Betacoronavirus. Bat coronavirus RaTG13 and SARS‐CoV (figure adopted from Mittal et al^15^ without modification)

The CoVs genome was 58 percent similar to that of the NSP‐coding area and 43 percent similar among different coronaviruses with a structural‐protein‐coding region of 54 percent at the entire genome level.^12^, ^18^, ^19^ It therefore highlights the need for rigorous monitoring of nsps and a greater propensity for structural proteins to respond to changing conditions. The genomes of RNA infections are typically smaller than 10 K nucleotides, as transformation rates in the replication of RNA infections are significantly higher than those in DNA infections. The CoV genome (∼30 kb) is the largest of all RNA pathogens, many times larger than that of the second‐largest RNA. CoVs of all RNA infections are familiar to 3‐5′ exoribonuclease, and they have been shown to act as an editing component of the RTC.^12^, ^20^, ^21^ Detailed studies have shown that the 2019‐nCoV has a similar coronavirus genome structure. It is also part of the beta coronavirus family that includes Bat‐SARS‐like (SL) ZC45, Bat‐SL ZXC21, SARS‐CoV, and MERS‐CoV. The phylogenetic CoV tree, 2019‐nCov, is associated more strongly with the bat‐SL‐CoV ZC45, bat‐SL‐CoV ZXC21, and more remotely with the SARS‐CoV^14^, ^22^, ^23^, ^24^ (Figure 2).

### 1.2 Coronavirus replication protein functions

The greater part of the nsps nsp1‐16 is responsible for their basic tasks in the replication of CoVs. The elements of some nsps are unclear or not properly identified. The virion assemblage and contamination of CoVs are at the core of four structural proteins. S‐protein homotrimers form the spike outside the infection particles and responsible for the hosts' attachment.^12^, ^17^, ^25^ There are three transmembrane spaces in the M protein. It forms virions, supports the structure of the membrane, and helps to adhere to the nucleocapside. The E protein is responsible for uniting and discharging the infection, and for pathogenesis it is essential. The N protein includes two areas where different methods can carry the infected RNA genome. The N protein will hold nsp3 protein so the replicate transcriptase (RTC) genome is preserved, and the embedded genome is packaged into viruses.^8^, ^13^, ^25^, ^26^ The N protein is also an antagonist for RNA interferon (RNAi) and virally encoded repressor (VSR), which benefit from viral reproduction. However, the known aspects of the 16 NSPS are outlined in the Table 1.

**TABLE 1:** Role of non‐structural proteins in the coronaviruses replication cycle

Non‐structural proteins of coronaviruses	Function
NSP2	MHV and SARS‐CoV genome RNA were created by deletions of the nsp2 coding sequence (MHVΔnsp2 and SARSΔnsp2, respectively), but its function is not known in nCoV.
NSP3	It binds to viral RNA, nucleocapsid protein, as well as other viral proteins, and helps in the processing of polyproteins. By de‐ADP‐ribosylation, de‐ubiquitination, and de‐Acetylation, innate immunity is counteracted by the host.
NSP4	Assembly of murine coronavirus DMVs
NSP5	Responsible for IFN signaling inhibition.
	Cleavage of 3CLpro, MPro
NSP6	Restrict autophagosome extension
NSP7	Cofactor with NSP8 and 12
NSP8	N/A Unknown
NSP9	Interacts with NSP8 and Replication of Corona virus.
NSP10	Scafold for NSP 14 and 16.
NSP11	The role of virus replication, but its exact functions and mechanisms of action are not well known.

### 1.3 Pathogenesis of COVID‐1

In humans as well as in animals, there are usually four (human coronaviruses 229E, NL63, OC43, and HKU1) coronaviruses responsible for the infection of the upper respiratory tract, and the impact is relatively mild. However, three coronaviruses can cause and reproduce fatal pneumonia in the lower respiratory tract (serious acute coronaviruses respiratory syndrome [SARS‐CoV], Middle East Coronvirus Repiratory System [MERS‐CoV], and SARS‐CoV‐2) are present. SARS‐CoV‐2 is a gene that produces beta‐coronavirus. SARS‐CoV is the closest relative of human coronaviruses, having a genetic resemblance of 79 percent. However, SARS‐CoV‐2 is most similar to the RaTG13 bat coronavirus in all known coronavirus sequences with a 98% similarity and also has a high similarity between the pangolin coronavirus (scaly ant eater) sequences. The transmission of SARS‐CoV‐2 is mainly through respiratory droplets, with a possible but not established fecal‐oral transmission route as in the case of other respiratory coronaviruses.^27^, ^28^

The median time of infection is approximately 4‐5 days before symptoms start and, within 11.5 days, 97.5% of symptoms develop symptoms. Patients with COVID‐19 typically have fever and dry cough at the time of hospital admission.

**FIGURE 3:**
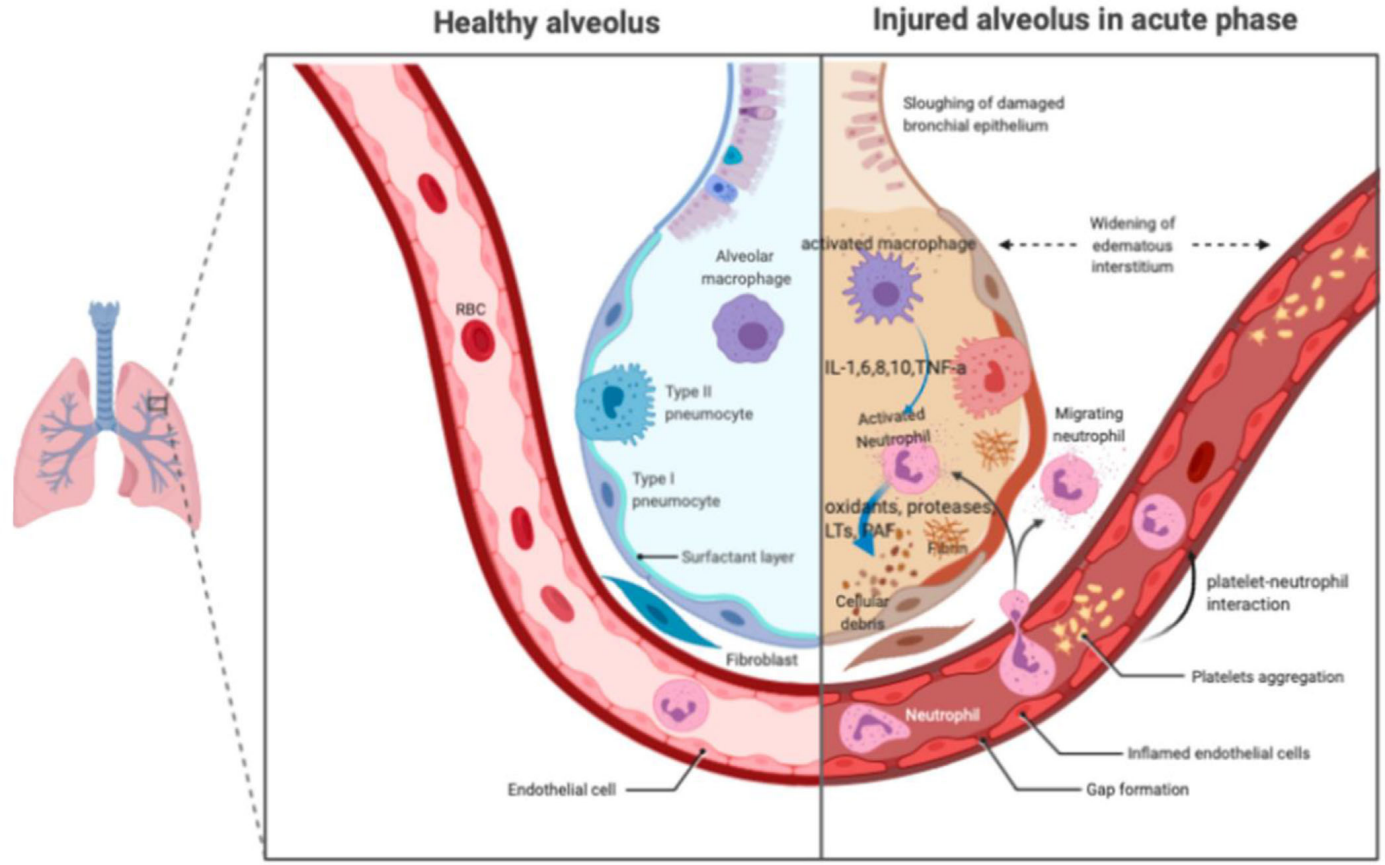
Acute respiratory disorder syndrome (ARDS) pathology in the acute period. Alveolar macrophages secrete cytokines in the air to induce chemotaxis and trigger neutrophils locally. Neutrophils can release oxidants, proteases, leucotrienes, and other proinflammatory molécules such as PAF (image adapted from^29^ without change) Abbreviations: IL, interleukin; LTs, leukotriene; PAF, platelet active factor; TNF, necrosis factor of tumors

The infection SARS‐CoV‐2 is very similar to the pathophysiological SARS‐CoV infection with violent inflammatory reactions closely linked to aerial damage. Therefore, not only the viral infection but also the reaction of the host, is the cause of the severity of the disease in patients. The trend towards increased severity with age is generally in line with the SARS‐CoV and MERS‐CoV epidemiologies. Acute respiratory distress syndrome (ARDS) refers to the diffuse damage caused by inflammation, mechanical stimulation, shock, blood transfusion and other factor to the pulmonary capillary endothelium and alveolar epithelium and is the key cause of poor prognosis in critical illnesses. Pathological changes linked to ARDS include primarily alveolar capillary membranes, increased permeability of the alveoli, epithelial and interstitial edema, pulmonary edema, disorders of the spread of carbon dioxide, and gas interchange. This condition leads to uncompromising hypoxemia, which inevitably leads to respiratory distress. A recent global epidemic of pneumonia caused by serious acute coronavirus 2 (SARS‐CoV‐2) disease varies from a symptomatic or mild high respiratory tract infection to extreme pneumonia, ARDS or even death. Furthermore, the extensive release of the cytokine immune system will lead to cytokine storm and septic disease symptoms that cause 28% death of fatal COVID‐19 cases in viral infections and/or secondary infections. In such cases, uncontrolled inflammation causes organ damage in the heart, hepatic, and renal systems by the organ failure (Figure 3).

### 1.4 Cytokine storm

**FIGURE 4:**
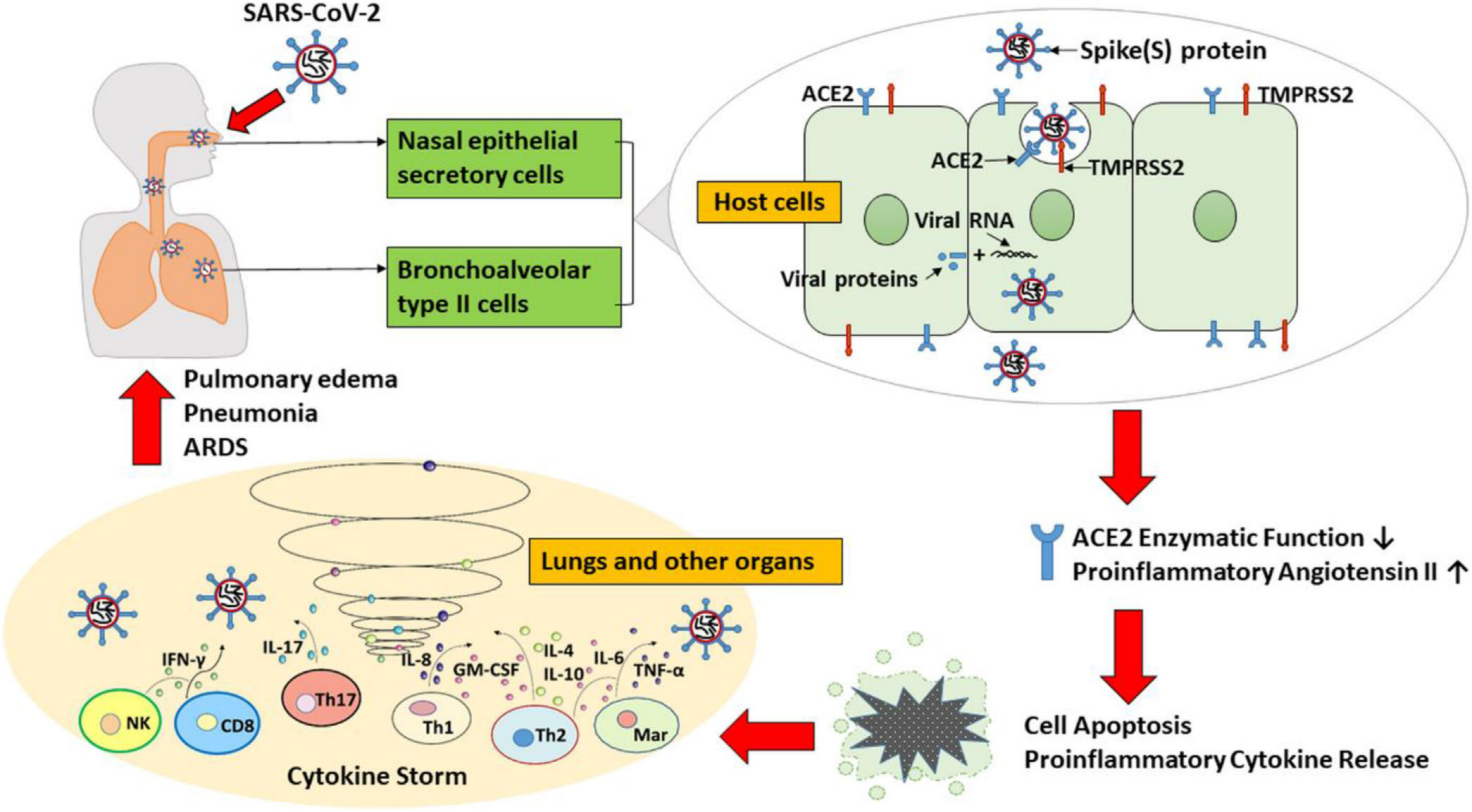
Schematic diagram of how COVID‐19 is triggered by SARS‐CoV‐2. The SARS‐CoV‐2 virus enters the nasal passages. The S‐Protein on the surface of the virus attaches to the secretory cells of the nasal epithelial and membrane protein ACE2, highly expressed in bronchoalveolar cells of type II. SARS‐CoV‐2 then reaches the host cell through phagocytosis. The enzymatic activity of ACE2 is diminished, or totally abrogated, and the amount of proinflammatory angiotensin II increased. High angiotensin II levels in the lung interstitium stimulate apoptosis, release proinflammatory cytokines, and cause inflammatory reactions, which result in cytokine storm symptoms and pneumonia in COVID‐19 patients and serious ARDS Abbreviations: ACE2, angiotensin‐convert enzyme 2; GM‐CSF, colony stimulating agent of granulocyte‐ macrophage; IFN, interferon; IL, interleukin; Mar, macrophage; NK, natural cell killer; TMPRSS2, transmembrane protease serine 2; TNF, colonial necrosis factor. (Reprinted with copyrights from Xiao et al^33^)

One of the widespread causes of death in the newly announced COVID‐19 pandemic appears to be cytokine storm. The infection is followed by an aggressive inflammatory action in the series of event known as “cytokine storm” where a significant amount of pre‐inflammatory cytokine is released. Host immune responses to the SARS‐CoV‐2 virus are hyperactive and lead to excessive inflammatory reactions. Several research in COVID‐19 patients examining cytokine profiles indicated that the cytokine storm associated directly with lung damage, multi‐organ failure and a weak pronouncement of extreme COVID‐19. The primary cause of COVID‐19‐related death is ARDS. The key immunopathology characteristics of SARS‐CoV, MERS‐CoV, and SARS‐CoV‐2 were identified in these ARDS. Cytokine storm is an unregulated reaction of the immune system characterized by a systemic, high‐chemiocin secretions (e.g. C‐X‐C cytokine motif 10[CXCL10], CXCL9, CXCL8, C‐C chemo ligand 5[CCL5], CCL3, CCL2, etc.) and pro‐inflammatory cytokines (e.g. alpha‐fctor, [IL‐33], IL‐18, interleukin interferon, gamma interferon, IL‐6, IL‐1b, gamma‐a, etc). In a human organ, a strong cytokine storm will lead to a violent attack by multiple organs and ultimately to the death from ARDS [Figure 4].^5^, ^30^, ^31^, ^32^

### 1.5 Potential therapeutic targets and their role in SARS‐CoV‐2 infection

It is time to look for the target proteins for a highly specific drug design and to evaluate the successful treatment of COVID‐19 medicines. The SARS‐CoV‐2 is of the same class as the SARS‐CoV and the MERS‐CoV and very similar to that used by the SARS‐CoV for viral proteins required for host cell entry and replication. SARS‐CoV and MERS‐CoV experiments are thus a strong source for identifying new therapeutic objectives in SARS‐CoV‐2. CoVs are in the nidoviral family Coronaviridae, they are enveloped and have a positive RNA genome. SARS‐CoV‐2 is the four genera (alpha, β, μm), which primarily consists of four structural proteins: Spike (S), envelope (E), membrane (M), and nucléocapsid (N). Two large target groups are present: one acting on the human immune system/cells, the other work for the coronavirus.^25^, ^34^, ^35^, ^36^

The human immune system regulates the coronavirus replication by blocking signalling pathways that indicate antiviral effect. Potential coronaviral therapy involves the prevention and replication of viral RNA synthesis, prevention of entry into the host cells, restoration of host immunity, and the blockade of structural proteins possible by careful targeting for genetic material, enzymes and functional proteins.^6^, ^31^, ^37^

Possible targets involved in virus RNA synthesis and replication are nsps (Table 1), which occupies approximately two‐thirds of the genome, play important role in RNA transcription, translation. Nsps also carry out protein synthesis, processing and modification, and virus replication. Nsps are considered key targets for possessing biological functions and active sites. Nsps consists of: 3C‐like main protease (3CLpro/Nsp5), Papain‐like proteinase (PLpro), RNA‐dependent RNA polymerase (RdRp/Nsp12), and helicase/Nsp13 that can be considered for the development of inhibitor.^38^ The research studies report the role of 3CLpro, also known as Nsp5, in cleavage of viral peptides into functional units required for virus replication. To produce mature enzymes 3CLpro is cleaved from poly‐proteins followed by downstream cleaving of Nsps to release Nsp4 to Nsp16.^4^, ^12^, ^13^, ^17^, ^39^

**FIGURE 5:**
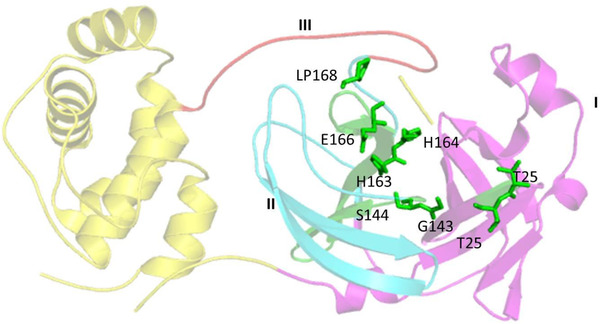
Representation of SARS‐CoV‐2 3CL, PDB ID‐6VYB,^16^ showing three domains: domain I in magenta color, domain II in cyan, and domain III in red color with active site residues represented in green colored sticks. Three dimensional structure of 3CL (PDB‐ID: 6LU7), retrieved from (Research Collaboratory for Structural Bioinformatics Protein Data Bank (RCSB PDB) represented in cartoon structure using molecular graphics system PyMol, Version 2.0 Schrödinger, LLC, where helices are drawn as helical cylinders, and beta sheets are drawn as arrow and are connected by loops

3CLpro can cleave 11 sites in the p1 position of PP1, it mediates maturation of Nsps, a critical step in the life cycle of the virus and highlighting its role as therapeutic target. There are three important domains in 3CLpro, viz; I (residues 8‐101), II (residues 102‐184) and III (residues 185‐200), constituting of active site (Cys145 and His41) present in the gap formed by domains I and II. Studies reveal important active sites in 3CL (PDB_ID: 6LU7) as T25, T26, V3, G143, S144, H163, H164, E166, and P168, as shown in Figure 5.

PLpro enters into the host cell and releases single‐stranded positive RNA, which is translated into the viral poly‐proteins by protein translation machinery of the host cell. PLpro is critically required for N‐terminus cleavages of the poly‐protein replicase to release Nsp1, Nsp2, and Nsp3, which are essential for the correction of virus replication. PLpro also serves as a deubiquitinase that can deubiquinate some host cell proteins contributing to immune suppression. PLpro sequences of SARS‐CoV‐2 has similar active site as that of SARS‐CoV, with sequence similarity of 83%, PLpro has striking feature of antagonizing host's innate immunity, and is vital for virus replication, therefore, with the above information it can be considered as a good therapeutic target. Currently, a PLPro inhibitor lopinavir is employed by the physicians in combination with anti‐HIV for COVID‐19 pateints, had shown positive effects.^40^, ^41^, ^42^

**FIGURE 6:**
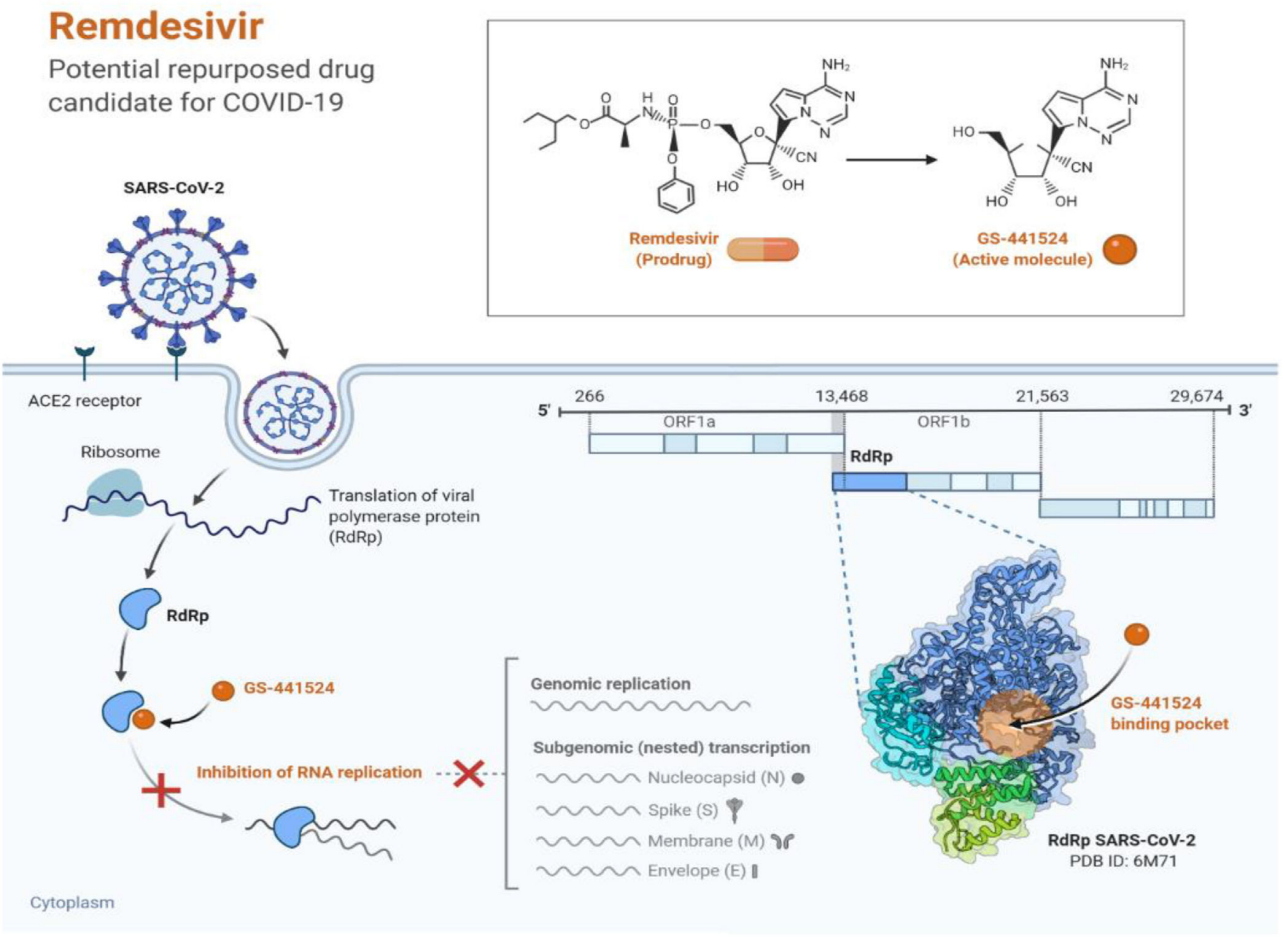
This figure template depicts the potential mechanism of action of Remdesivir against coronavirus replication. It also depicts a 3D structure of the proposed binding pocket of RdRp polymerase on the right. Created with BioRender.com (Adapted from BioRender.com without modification)

RdRp also known as Nsp12, is also required for virus replication/transcription. RdRp is involved in the synthesis of full‐length negative‐strand RNA, which is used by RdRp in production of viral genomic RNA. SARS‐CoV and SARS‐CoV‐2 share sequence similarity of 79% at the genome level, while RdRp and 3CLpro protease of SARS‐CoV‐2 share sequence similarity of more than 95% with those of SARS‐CoV. The polymerase RdRp domain is positioned at the C‐terminus and has a conserved motif: Ser‐Asp‐Asp. Nsp8 is capable of synthesizing up to six nucleotides that can be used as a primer for Nsp12‐RdRp RNA synthesis, also Nsp7_Nsp8 complex increases the RdRps enzyme activity of Nsp12 by binding Nsp12 to RNA, rendering Nsp12‐RdRp as an important target. For example, Remdesivir is a nucleotide analogue imitating the adenosine structure. It was originally created to treat Ebola by Gilead Sciences, Inc. In Ebola, remdesivir has been found to act as a binding substrate of RNA‐dependent RNA (RdRp) which substitutes ATP for polymerization before this process is finalized, also known as a "chain‐terminator." The active form of remdesivir was hydrolyzed and decorated with triphosphates and used as a nucleoside the core of redesivir. This hydrolyzed and phosphorylated remdesivir is named "RemTP." Redesivir can, like other nucleotide analogs, be used as a wide‐spectrum antiviral drug due to the structural similarity of RdRp's with different viruses. It was clinically tested and demonstrated substantial efficacy against MERS‐CoV. The prodrug (Remedesivir) in its active form GS‐441524 is now being currently studied in its phase III trial in both USA and European countries.^3^, ^15^, ^43^, ^44^, ^45^ The exact mechanism of the Remedesivir is schematically presented below in Figure 6.

**FIGURE 7:**
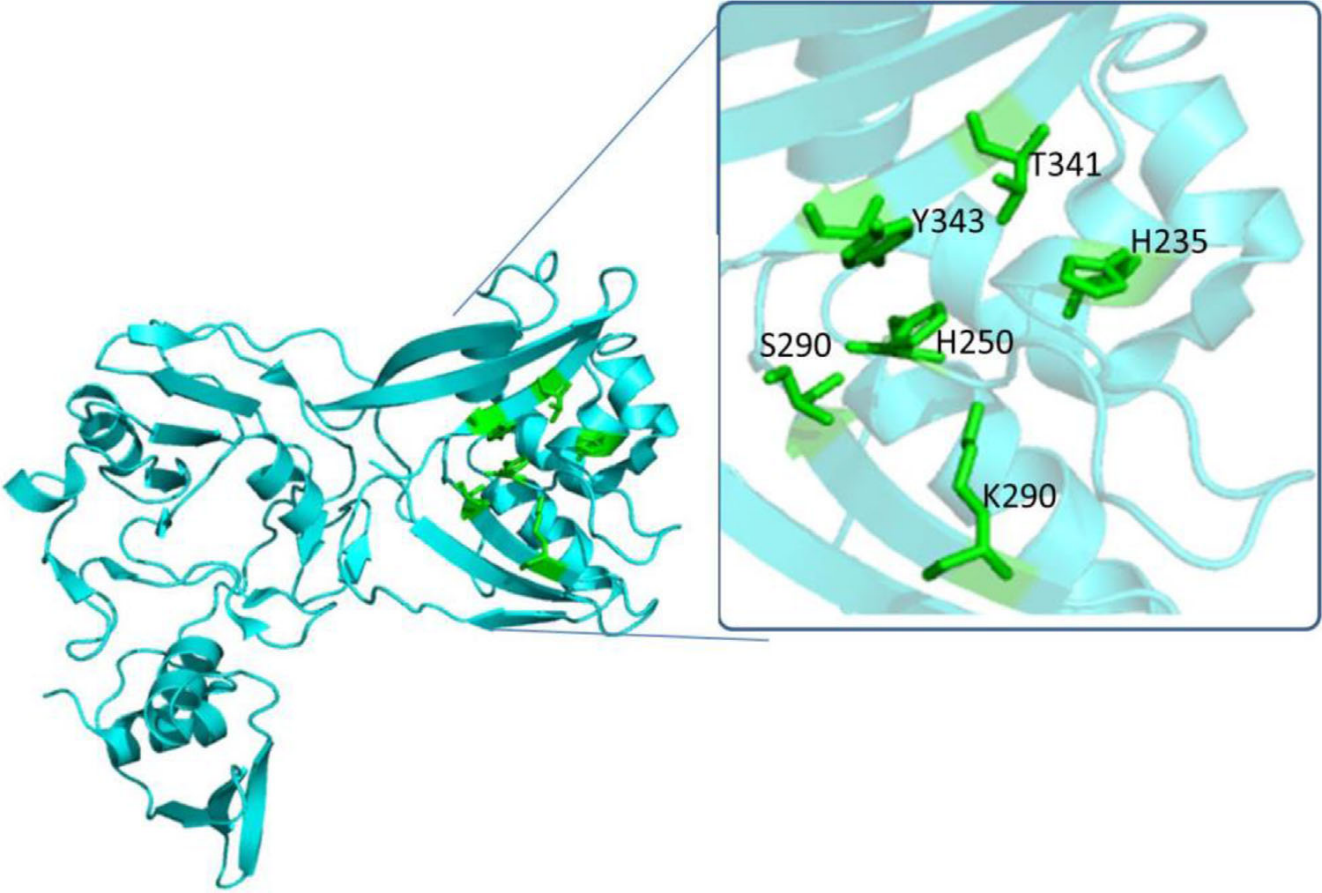
Cartoon representation of SARS‐CoV‐2 NSP15, PDB ID‐6VWW in cyan color focusing at the active site residues marked with green colored stick representation.^46^ The figure presents three dimensional structure of NSP15 (PDB ID‐6VWW), retrieved from Research Collaboratory for Structural Bioinformatics Protein Data Bank (RCSB PDB) represented in cartoon structure using molecular graphics system PyMol, Version 2.0 Schrödinger, LLC, where helices are drawn as helical cylinders, and beta sheets are drawn as arrow connected by loops

Helicase, also known as Nsp13, a multi‐functional protein contains two domains: N‐terminal binding (MBD), consisting of a 26‐cysteine residue to form a Zn binding domain and a C‐terminus‐conserved helicase (Hel) domain. Studies show the importance of the highly preserved and essential SARS‐Nsp13 in the replication process. Other important Nsps to synthesize RNA viruses and replication for therapeutic purposes can be considered: Nsp3b, Nesp3e, Nsp7 Nsp8, Nsp9, Nesp10, Nsp14, Nsp15, and Nsp16. Nsp15's C‐terminal N endo U terminal domain plays a catalytic role, composed by H235, H250, K290, T341, Y343, and S294 as shown in Figure 7.

**FIGURE 8:**
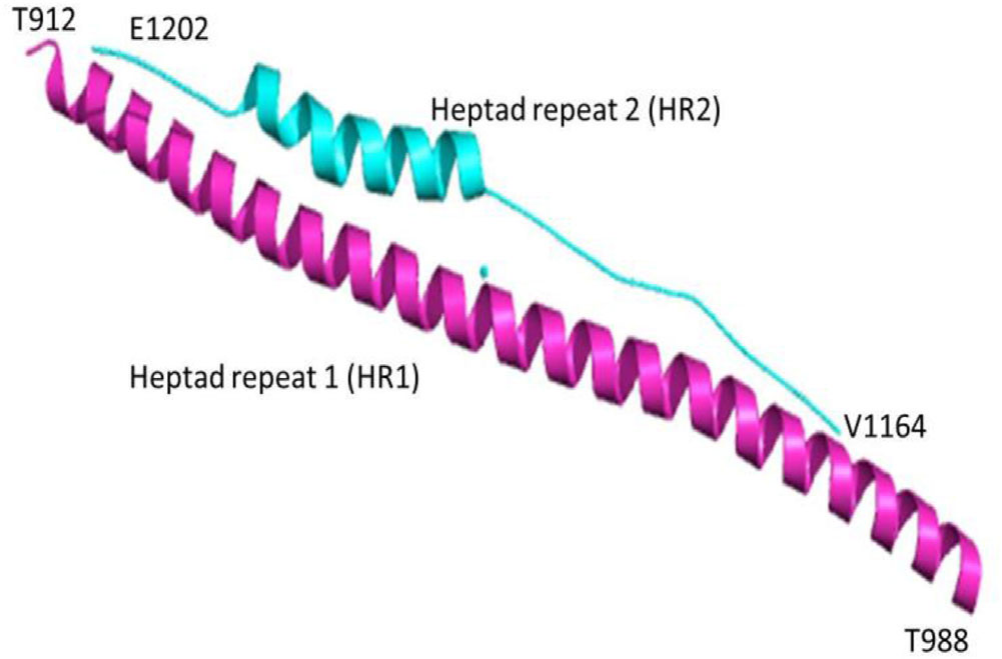
The SARS‐CoV‐2 S (PDB ID:6XLT) sub‐unit domains HR1 and HR2 are shown in cartoon pictures. The HR1 domain is shown in magenta and the HR2 domain in cyan. The figure presents three dimensional structure HR1 and HR2 (PDB ID:6XLT), retrieved from Research Collaboratory for Structural Bioinformatics Protein Data Bank (RCSB PDB) represented in cartoon structure using molecular graphics system PyMol, Version 2.0 Schrödinger, LLC, where helices are drawn as helical cylinders, and different domains are colored in magenta and cyan

The glycosylated Spike (S) protein of I‐TM type is another important viral structural protein that activates host immune responses.^47^, ^48^ The S‐protein assembles the surface of the virus as a cutter into a corollary structure and controls host cells' invasion both SARS‐CoV and SARS‐CoV‐2 via an enzyme 2 (ACE2) binding to the host cell membrane of the receptor protein.^30^, ^49^, ^50^ The invasion process involves the primation of S protein that is mediated by the host‐supplied serine protease TMPRSS211. TMPRSS211 cleavates S protein into S1 and S2 for S1 to bind to host surface cell receptors, and S2 mediates virus‐cell‐cell membrane fusion. S1 subunit S protein receptor‐binding domain (RBD) binds with an ACE2 receptor in the target cell, with two more essential domains: heptad‐repeat 1 (HR1) and the 2 (HR2) domains in the S2 subunit S protein interact to form a six‐HB fusion center to close the virus and cell membranes, thus raising the likelihood of fusion or infection. S1 subunit (residues 14‐685), located within the N‐terminal, constitutes N‐terminal domain, RBD, and the RBM motif. The S2 sub‐unit contains fusion peptides, repeat heptads 1 (HR1, 910–988 residues), repeat heptads 2 (HR2, 1162‐1206 residues), transmembrane domain, and cytoplasmic domain. RCSB Protein Data Bank Structure of HR1 and HR2 (PDB ID‐6XLT A)^51^ as shown in Figure 8.

**FIGURE 9:**
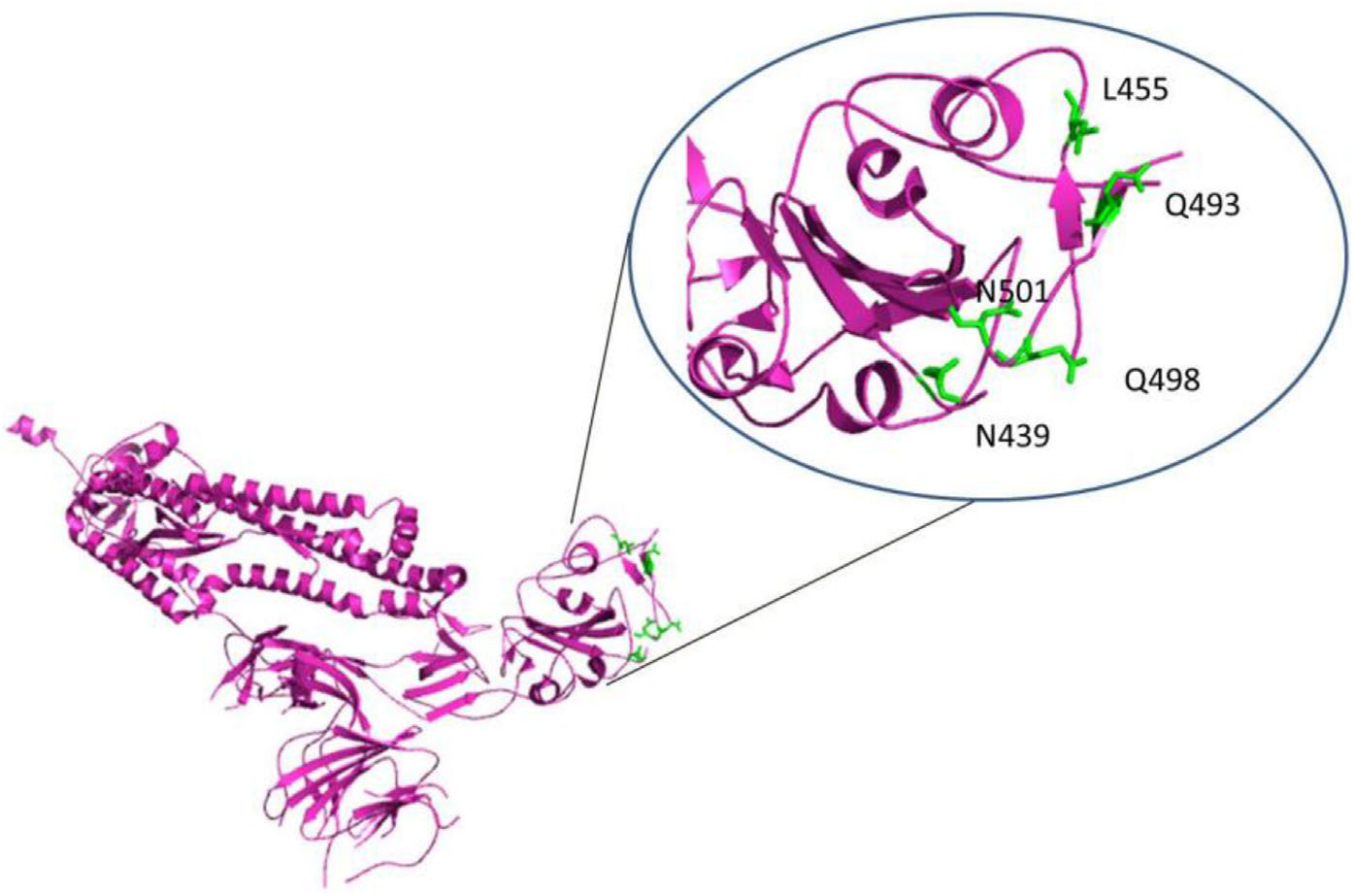
Cartoon representation of SARS‐CoV‐2 S protein, PDB ID‐6VYB. (A) Partially open structure of SARS‐CoV‐2 in magenta colored cartoon, (B) Conserved residues involved in binding with ACE2, as shown in green colored sticks. Three dimensional structure of SARS‐CoV‐2 S (PDB_ID:6VYB), retrieved from Research Collaboratory for Structural Bioinformatics Protein Data Bank (RCSB PDB) represented in cartoon structure using molecular graphics system PyMol, Version 2.0 Schrödinger, LLC, where helices are drawn as helical cylinders, and beta sheets are drawn as arrows connected by loops

Six essential residues retained for ACE2 binding are N439, L455, F486, Q493, Q498, and N501, as shown in Figure 9. Spike structural integrity and cleavage activation play an important role in virus invasion and virulence. For the production of anti‐viral drugs, therapeutic strategies to prevent coronavirus reaching host cells by targeting special receptors on the host surface with S proteins, specifically fusion machinery of SARS‐CoV‐2 are invaluable. E protein (E‐channel) offers essential biological features for structural integrity and host virulence of coronavirus.^52^ For successful N protein linkage with coronavirus RNA in household cells, coronavirus N (N‐terminal RNA binding domain [NRBD] and C (C‐terminal RNA binding domain [CRBD]) are needed, and E protein or N protein can be used as a therapeutic target in anti‐virus drug discovery.^6^, ^41^, ^53^

Other relevant goals are the factors associated with coronavirus virulence, Nsp1, Nsp3c, and ORF7a that interfere with host's inborn immunity and aid with coronavirus immune escape. In addition, host‐specific receptors or enzymes can be considered essential targets for discovery of medicines. The linkage of viral S protein with its ACE2 receptor in host cells and potential viral endocytosis in cells may also be a promising drug target. The host cell produces TMPRSS2, used for proteolytic processing of S protein priming in human cell binding receptor ACE2, which can be demonstrated to be a possible drug target.

Table 2 lists classification of the potential therapeutic targets, their key function in viral infection, existing drugs or drug candidates that are reported to block similar viruses and other predicted inhibitors of SARS‐CoV‐2 based on virtual screening of small molecules from Zinc database.

**TABLE 2:** Key target proteins, their function, potential inhibitors, and predicted inhibitors

Target proteins classification	Target protein	Function	Drug candidate	Expected drug candidate
Virus RNA synthesis and replication	Papain‐like proteinase (PLpro)	Proteolysis of viral polyprotein into functional units	Lopinavir	Ribavirin, β‐thymidine, valganciclovir, oxprenolol, aspartame, doxycycline, chloramphenicol, acetophenazine, riboflavin, iopromide, reproterol, 2,2′‐cyclocytidine, chlorphenesin carbamate, nicardipine, silybin, sildenafil, dantrolene, sulfasalazine.
	3C‐like main protease (3CLpro)	Proteolysis of viral polyprotein into functional units	Lopinavir	Carminic acid, mimosine, flavin mononucleotide, lutein, cefpiramide, phenethicillin, candoxatril, nicardipine Estradiol valerate, pioglitazone, conivaptan, telmisartan, doxycycline, oxytetracycline.
	RNA‐dependent RNA polymerase (RdRp)/ NSP12	Replication of viral genome	Remdesivir, Ribavirin	Valganciclovir, chlorhexidine, ceftibuten, fenoterol, fludarabine, itraconazole, cefuroxime, atovaquone, chenodeoxycholic acid, cromolyn, bromide, cortisone, tibolone, novobiocin, silybin, idarubicin, bromocriptine, diphenoxylate, benzylpenicilloyl g, dabigatran etexilate.
	Helicase (Nsp13)	Virus replication		
Virus structural proteins Virulence factor	viral spike glycoprotein (S protein)	Viral surface protein for binding to host cell receptor ACE2		Arbidol,chloroquine
	Transmembrane protease, serine 2 (TMPRSS2)	A protease from host cells that primes S protein to make its binding easier to ACE2		Camostat mesylate
	E protein (E‐channel)	Bind N proteins with coronavirus RNA in host cells		
	Nsp1	Alter innate immunity of the host and assist in the immune escape of coronavirus		
	Nsp3c	Alter innate immunity of the host and assist in the immune escape of coronavirus		
	ORF7a	Alter innate immunity of the host and assist in the immune escape of coronavirus		
Host‐specific receptor or enzymes	angiotensin‐converting enzyme 2 (ACE2)	A viral receptor protein that binds with viral S protein in host cells		Arbidol
	angiotensin AT2 receptor	An significant factor in blood pressure and cardiovascular system volume control		L‐163491

### 1.6 Cytokines therapy

The infection caused by the COVID‐19 leads to the production of various cytokines by the host system in order to eliminate its propagation. The effective cytokines released in effect of the infection are interferons (IFNs), interluekins (ILs), and lymphokines. On the basis of their binding and forming receptor complex for signalling as well as their sequence homology, they can be classified into three types: type I (IFN‐α, IFN‐β, IFN‐k, etc), type II (IFN‐ϒ), and type III (IFN‐∆).^41^, ^53^, ^54^, ^55^ As, the cytokines interfere with the viral proteins and affect the replication process, they can be utilized for the therapeutic purpose. Human serum albumin in combination with IFN has been used for wide range of viral infections including SARS. The combination with HSA allowed extended plasma half‐life from 10 hours to 12 days and thus allowed prolonged effect of IFN. Variants of IL‐28A and B, along with IL‐29 have been effectively used for the SARS infections. Another study revealed the recombinant HIFN‐ω has potential in inhibition of SARS viral activity. Thus, the similar strategy can be employed to address therapeutic routes for treating coronavirus.

### 1.7 Vaccine development and role of nanomedicine against CoVs

It is pivotal to create successful immunizations to control the COVID‐19 pandemic. The development of a vaccine against SARS‐CoV‐2 requires a detailed understanding of how this novel coronavirus, developed its viral structure as well as the targets of vaccines that have been developed against related viruses, such as SARS‐CoV. The detailed study of SARS‐CoV2 virus had revealed its homology with two other deadly coronaviruses, SARS and MERS, the vaccine recognized in these infections might encourage the plan of new vaccine against SARS‐CoV2.

In our previous section we have discussed about the coronavirus structure, which helps to understand the molecular structure. The experimental studies have shown that viral S protein subunit vaccines produce large antibody titer there may not be a formally approved and higher protection than attenuated SARS‐CoV and DNA‐based S protein vaccine. For widespread use against SARS‐CoV or MERS‐CoV, there have been a number of targets identified that may be useful in the development of a vaccine against SARS‐CoV‐2.^55^, ^56^ There have been trials involving inactivated viruses lacking the E protein, which has been shown to remove the virulence of coronaviruses and is a potential alternative. DNA vaccines involving the N protein are not considered effective, although there is some evidence that co‐administration with the M protein can augment this effect. The Mark Denison group had revealed that mutation into the Orf1a/b protein can completely attenuate the virulence in mice models and inhibited the replication of the Virus.^32^, ^57^ In another study, Tzyychoou has developed gene delivery with gold nanoparticles and showed that vaccination of mice nucleocapsid fusion (N) proteins lead to nucleocapsid‐specific humoral and T‐cell response.^58^, ^59^ David B Weiner, in March 2020 developed DNA‐based vaccine comprised of MERS‐CoV protein, showed enhanced humoral as well as cellular immune response.^60^

In this pandemic season, a large group of scientists has turned their concentration to prevent its spread. As of now, there is no particular treatment accessible for COVID‐19. In the meantime, among different fields of science and innovation, nanotechnology can be of high potential in the prevention, diagnosis, and treatment of COVID‐19. Several different candidates of the vaccine have been formed with nanocarriers since the SARS‐CoV outbreak in 2002 (Table 3). Lipid‐based NPs, such as liposomes or solid‐lipid NPs (SLNs), show high similarities to virus particles that mimic a similar structure of surroundings such as viruses, which can also be chemically adjusted.^61^ Although liposomes in NPb‐V are restricted to respiratory viruses, the use of these systems is highly advantageous in vaccine production because carriers can be altered without virus‐specific peptide usage in order to achieve similar characteristics as viruses.^62^ Similarly, viral‐like particles (VLPs) are based on viral proteins, in particular viral capsid proteins, which assemble in 20‐200 nm spherical nanocarriers. Such VLPs were successfully tested for their influenza and RSV vaccination capability. For example, VLPs made out of A / PR8/34(H1N1) hemagglutinin and matrix (M1) have been examined for their immunogenic capacity in influenza after nasal administration to mice.^63^, ^64^, ^65^ In addition, the capacity of exosomes for the supply of RNA and DNA for vaccination purposes has recently been demonstrated.^66^, ^67^ In addition, exosomes can be modified to express viral antigens, similar to liposomes. In 2007, it was shown that 293T‐cells are transfected by S‐protein‐expressing plasmids and result in S‐protein exosomes. Animal studies using mice BL57/6 showed neutralizing levels of antibodies after treatment, demonstrating clearly how exosomes can be used as NPb‐V, However, because cells produce exosomes, the quantities that can be manufactured are small.^68^, ^69^ Nanovaccines has a great potential in prophylactic and therapeutic approaches to enhance the antigen presentation and processing and as an immunostimulatory adjuvant. With the growing interest in RNA and DNA‐based vaccines, the combination of RNA and DNA‐based vaccines is becoming an interesting strategy for overcoming limitations of the current vaccines. Combining RNA with nanocarriers is an effective way to deliver small interfering RNA (siRNA) for the treatment of a number of disorders, like cancer, infections, autoimmune, and neurological diseases.^70^, ^71^ For example RNA‐based vaccine mRNA‐1273 is based on the combination of the RNA‐based drug, and a Nano carrier system can be used for the delivery of antigens that prevent premature body degradation and enable the molecules to convert into functional immunogens to avoid possible side‐effects.^61^, ^72^

**TABLE 3:** Overview of different nanoparticle‐based vaccines for coronaviruses

Nanocarrier	Characteristics	Target	Therapeutic component	References
Liposome	Recombinant S1 subunit on the exterior of liposomes, encapsulation of TLR4 and TLR9 agonist	SARS‐CoV2	S1 subunit of the virus; adjuvants: amphiphilic adjuvant monophosphoryl lipid A for TLR4 and CpG oligodeoxynucleotide for TLR9	^84^
Iron‐oxide nanoparticles (IONP)	Docking study done to show interaction of Fe_2_O_3_ and Fe_3_O_4_ with the spike protein receptor binding domain (S1‐RBD) of SARS‐CoV‐2.	SARS‐CoV‐2	IONP	^85^
Gold NP	Can act as adjuvant and antigen carrier for the spike protein.	SARS‐CoV	Gold	^86^
Chitosan NP	Biotinylated chitosan NP for intranasal delivery, targeting dendritic cells.	SARS‐CoV	Nucleocapsid (N) protein of SARS‐CoV as vaccine Antigen	^87^, ^88^
PLGA polymer	Biocompatible, capsid‐like hollow, pH responsive nanoparticulate system.	MERS	STING agonist	^89^, ^90^

NP‐based vaccine system (NPb‐V) can be characterized into two main stratagems:

1. NPb‐Vs, when nanocarriers encapsulate antigen or RNA / DNA and
2. When the antigen is attached to the nanocarrier surface.

In the nanocarriers, antigens or RNA / DNA vaccines are primarily encapsulated to protect antigens from proteolytic degradation and allow APCs to be targeted.^73^, ^74^ These APCs process antigens induced into the cell surface or transfer infused RNA or DNA into the respective antigen until they are placed on the surface. This may be preferable when contrasting RNA with DNA, since RNA can be translated directly into the cell cytoplasm, while DNA need to first enter the target cell nucleus. A promising approach to encapsulation into nanocarriers of RNA or DNA can be applied to build NPb‐V against CoVs.

During clinical trials, various vaccines against RNA or DNA are being studied, making a promising strategy to combine these ribonucleases with nanocarriers. NPb‐V based on antigenic encapsulation is also likely to have a local depot effect, which extends the exposure of immune cells to the antigen.^75^, ^76^ The second strategy for NPb‐V is to directly attach antigens to the nanocarrier surface.^77^ The NPb‐V is therefore not intended to move a cargo to APCs, but to mimic the virus itself. The presentation of S‐protein antigens on top of a nanocarrier may, for example, cause a certain immune response to these antigens and thus form a promising CoV strategy. In particular, in combination with nanocarriers, the purification of immunoglobulins from patient plasma could create promiscuous NPb‐V against CoV.^78^, ^79^, ^80^ This strategy had many advantages over traditional vaccine design‐like weak immunogenicity, in vivo instability, toxicity issue, and their route administrations. The nanosized vaccine helps in the development of increased humoral and cellular immune response and increases the phagocytic uptake. The modifications of the nanoparticles with targeting ligands (antibodies, peptides, and carbohydrates) for the active targeting of immune cells and thus the development of specific and selective immune response. Another advantage of nanoparticles in vaccine delivery is that their small sizes of nanometer range are approximately the same as that of bacteria and viruses that are readily identified by the immune cells. The modification of the NPs surface allows the higher uptake of the therapeutics and thus affecting their dose inside the cells. Similarly, the nanoparticle size is another factor that affects their cellular uptake, like nanoparticles in a size range of 50 nm are optimum for non‐phagocytotic cells. There is an expectation that nanoparticles can roll out an improvement due to their small size and improved properties. The structural studies of coronavirus uncover its similarities with the nanoparticles. The modification of nanoparticles that allow their binding with the spike protein of the virus and irradiating them with the electromagnetic radiation mainly IR, can lead to its destruction and ultimately lead to the suppression of viral multiplication.^81^, ^82^, ^83^

Virus‐like nanoparticles (VNPs), due to their immunogenic composition that comprises viral membrane protein, help in the generation of higher antibody titre and generation of potent immune response when administered with an appropriate adjuvant. VNPs developed from the MERS‐CoV induced a higher neutralizing antibody response in mice models. Thus, the VNPs can be employed to develop a COVID‐19 vaccine candidate targeting the S protein of SARS‐CoV‐2 with adjuvant Matrix‐M.^64^, ^91^, ^92^

### 1.8 Nanomedicine techniques for targeting the immune system

Nanocarriers are now promising to provide anti‐inflammatory drugs to immune cells to prevent the virus from spreading across the body. Different forms of nanocarriers can be aimed for provoking an immune response to present immune‐causing antigens such nanocarriers are targeted at delivering anti‐inflammatory macrophages and T cells mainly involved in CRS and at blocking IL‐6, IL‐1, TNFα, and other cytokines.^93^ Similar therapies can be helpful in the treatment of cells infected with CoV. For example, Tocilizumabe, an IL‐6 antagonist, was loaded into SLNs for lung delivery combined with hyaluronate‐gold NPs to treat rheumatoid arthritis.^94^

Although numerous strategies have been developed for macrophage targeting over the last few years, pro‐inflammatory macrophages remain difficult to target, and studies that concentrate macrophages with the CRS background are rare.^93^ Nevertheless, the use of nanocarriers to target pro‐inflammatory immune cells have been demonstrated by different groups. The presence of mannose receptors on the surface of these macrophages (also known as CD206) is a promising objective for targeted delivery to macrophages. For example, mannose‐based bio‐reducible cationic polymers were produced, which facilitate the presence of a cellular absorption mannose receptor.^95^ These carriers were intended for producing siRNA in macrophages against TNFα expression which eventually reduced the reactions of pro‐inflammatory bowel disease. Mannose receptor is not only expressed in inflammatory macrophages but has disproportionate expression in anti‐inflammatory macrophages, which illustrates a problem with the precise targeted delivery of macrophage therapy — pro‐inflammatory and anti‐inflammatory marks are possible to share the configuration of unique nano‐carrying targeted markers.^77^, ^95^

### 1.9 Clinical trials for nanoparticles targeted for CoV‐2

Quick research and speedy profiling of the nanoparticles intended to be used for CoV‐2 are the key steps for a successful treatment of the disease. Although several nanoparticles are designed, only a few were able to make it to the clinical trials. Most of the clinical trials are failing because of no significant effect observed in the COVID‐19 patients.^96^, ^97^ Of the noted ones is the cholesterol‐rich non‐protein nanoparticle (MTX ‐LDE) encapsulating methotrexate for COVID‐19 called the Nano‐COVID 19 (NCT04610567, NCT04352465). Another such study is the evaluation of SARS‐CoV‐2 recombinant spike protein nanoparticle vaccine (NCT04368988) which is in the phase 1‐2 stage. Inhaled nanoparticle formulation of Remdesivir (GS‐5734) and NA‐831 (NEUROSIVIR) is under evaluation in Phase 1 stage that (NCT04480333). These trials show that although there are extensive efforts in place to combat CoV‐2 infections with nanoparticles, there is still a long way to reach a successful treatment regimen.

### 1.10 Nanomaterials for the detection of SARS‐CoV‐2

The fight against SARS‐CoV‐2 provides a wide range of techniques, including nucleic acid testing, mainly by means of the polymerase reaction chain (PCR) and serological tests to detect respiratory infection.^98^, ^99^, ^100^ A fast and reliable colorimetric bioassay has recently been established with the modification of plasmon gold nanoparticles (AuNP) using the specific antisense oligonucleotides designed for two SARS‐CoV‐2 regions of the N gene region.^101^, ^102^

**FIGURE 10:**
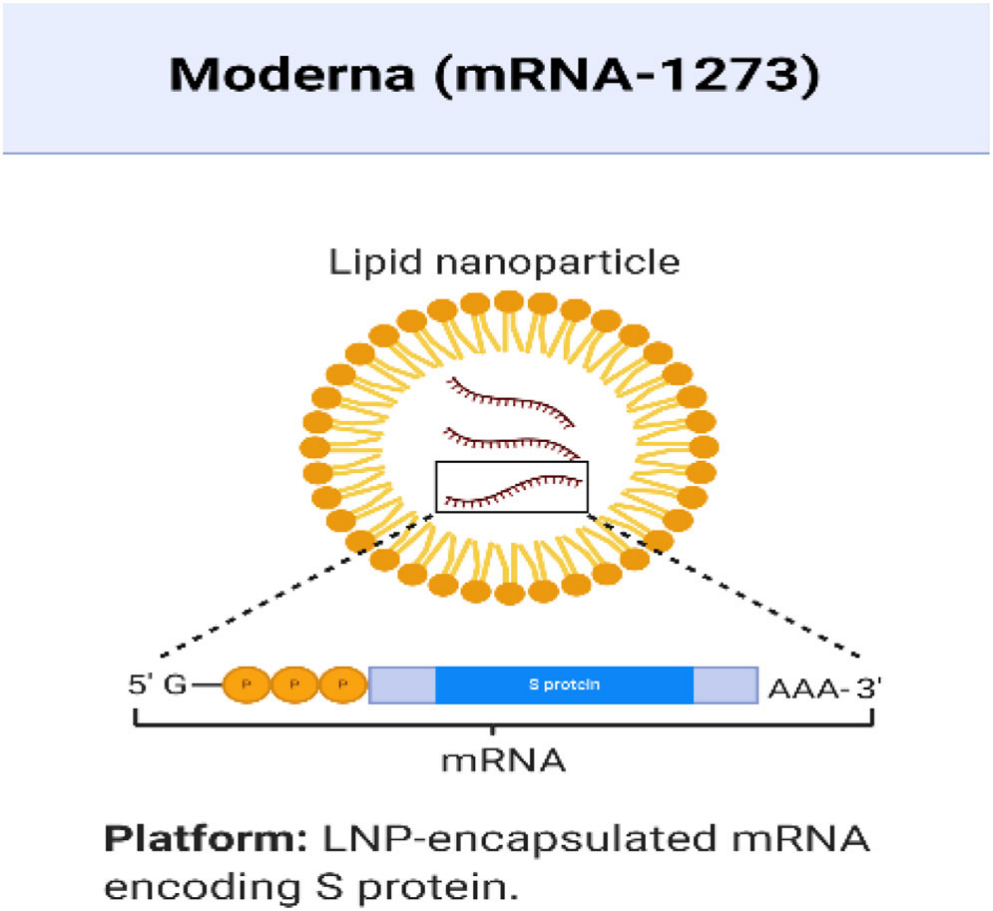
LNP‐encapsulation mRNA encoding S Protein (created with BioRender.com)

**FIGURE 11:**
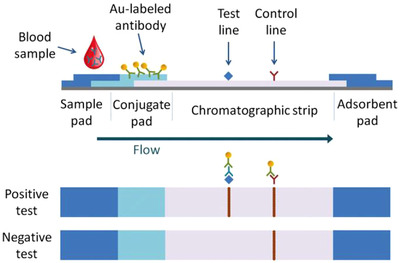
Schematic representation of an immunochromatographic blood or serum antibodies test, using AuNP lable for direct visualization (adapted from the advanced healthcare materials^105^)

Nanoparticles usually identified in ICT, also referred to as LFIA, mainly for the identification of antigens or antibodies. The advantage of employing NPs approach is that they might be handy for diagnosis when laboratory facilities are not available because they are easy to use, do not require trained personnel, can work even on small sample quantities (around 10‐20 μL), and generate rapid results in less than 20 minutes.^103^ As shown in Figure 10, the ICT normal configuration is: (a) sample pad and sample buffer; (b) combination pad with antibodies or antigens with a colloidal AuNp (c) Au‐labeled molecules bind to an antibody in the sample via a capillary action through a chromatographic strip to the test and contour as shown in Figure 11. Lack of color on the test line indicates that the target antibodies are not present in the blood sample. In the test line, antigens of S, M, and N protein SARS CoV‐2 are immobilized to detect IgM and IgG unique to this coronavirus.^104^ Li and colleagues recorded a new ICT configuration with two test lines that could simultaneously detect IgM and IgG, with a sensitivity of 88.7 percent and a specificity of 90.6 percent assessed in the same test in 15 minutes.^78^

For the detection of nucleic acids, immunochromatographic tests may also be configured. Broughton and colleagues reported the detection by CRISPR, a powerful gene‐editing tool, of viral RNA extracts from nasopharyngeal swabs, together with a lateral flow test that uses the AuNP label.^106^, ^107^, ^108^

This method has a high sensitivity for SARS‐CoV‐2 identification without cross‐reactivity for related coronavirus strains. Similarly, a portable built‐in microdevice that combines RT‐PCR and ICT was developed for the H1N1 colorimetric detection was reported a few years ago. This technology could also currently be useful for the detection of SARS‐CoV‐2.^109^, ^110^

**FIGURE 12:**
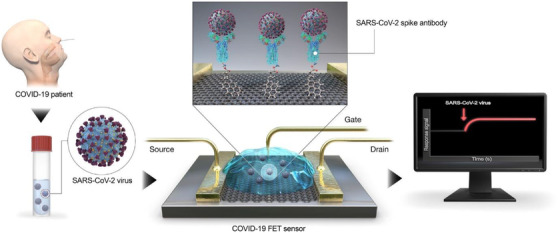
Schematic diagram of the activity of the COVID‐19 FET sensor. Graphene is selected, and SARS‐CoV‐2 spike antibody is combined with 1‐pyrenebutyric N‐hydroxysuccinimide ester, an interfacing molecule as probe connector, in the graphene layer (Reprinted with copyrights from Seo et al^98^)

To date, numerous biosensor platforms were established as SARS‐CoV‐2 diagnostic tools. Biosensors are the devices that use biochemical reactions to transform them in electrical, thermal, or optical indicators into observable readings.^98^, ^111^ The key parameters used in the design of SARS‐CoV‐2 biosensors are target analysis (viral RNA, antimicrobial agents or antibodies), nuclear acid receptor, antifoulant etc. The newly developed FET‐based bio‐sensor platform for the rapid detection of SARS‐CoV‐2 was recently reported by SEO and collaborators.^98^, ^112^ This biosensor is made of nanographene as a transducer and sensor material and a SARS‐CoV‐2 spike antibody added as a biomolecular receptor to the graphene sheet (Figure 12). The output of the bio‐sensor has been evaluated to enable rapid and highly reactive detection of SARS‐CoV‐2 in clinical samples of patients with antigenic protein, body virus, and nasopharyngeal swab. The antigen protein SARS‐CoV‐2 and MERS‐CoV were both highly specific and distinguished.^63^, ^113^

## 2 CONCLUSION

The novel SARS‐CoV‐2 has a rapid spread and high incidence which had given rise to unparalleled scientific effort to progress new vaccines or therapeutic agents to prevent or treat COVID‐19 disease. Several challenges still remain, however, in developing an effective SARS‐CoV‐2 treatment and in preventing future potential emergencies of new CoVs. In the recent past, different studies have been focused on developing effective vaccines, and several companies are currently working and evaluating various types of nanocarriers vaccines. The time required for meeting all criteria to approve the vaccines is one of the main obstacles in their development. Although a few weeks after the composition and genetic sequence of SARS‐CoV‐2 were known, the first vaccine candidates could have been designed, it might take months to more than a year before a candidate can reach the marketplace. While, in the current situation, the period of development and acceptance, is significantly shortened because SARS‐CoV‐2 infections need to be prevented, this period leads to repeated outbreaks and highlights of viruses, eventually rising the number of patient deaths.

Despite the enormous effort to develop a SARS‐CoV‐2 vaccine, there is no doubt that another CoV outbreak will occur in the future. As illustrated in Table 1, CoV ’s S protein is currently the focus of its use as an antigen, however, another virus structural protein could also use an antigen and provide a firmer vaccine than the S protein.

Overall, nanomedicine provides a valuable platform to further study the virus actions and to identify possibly unknown secondary target sites. As already described, VNPs, liposomes can imitate the behavior of the virus, employing nanocarrier will help in studying their kinetics and spread to the body. In order to achieve successful long‐term treatment for CoV infections and to form an important foundation for preventing or dealing with future CoV infections, interdisciplinary approaches are required. Furthermore, nanomedicine‐based strategies with already promising applications in SARS‐CoV, MERSCoV, may also be applied in SARS‐CoV‐2. Since the SARS‐CoV‐2 data are increasing quickly and changing, and some definitions and assumptions in this analysis might change due to new and more comprehensive insights into the viral characteristics and properties. In this study, some ideas and hypothetical definitions may need to be adapted on the basis of new findings and possible long‐term consequences of SARS‐CoV‐2 infection. The continued development and new insights into SARS‐CoV‐2 mechanisms and associated disease patterns will simplify the fast development of new therapies by offering a number of promising nanomedicine treatment platforms.

## CONFLICT OF INTEREST

The authors declare that there is no conflict of interest.
